# Mapping the Genetic Architecture of Gene Expression in Human Liver

**DOI:** 10.1371/journal.pbio.0060107

**Published:** 2008-05-06

**Authors:** Eric E Schadt, Cliona Molony, Eugene Chudin, Ke Hao, Xia Yang, Pek Y Lum, Andrew Kasarskis, Bin Zhang, Susanna Wang, Christine Suver, Jun Zhu, Joshua Millstein, Solveig Sieberts, John Lamb, Debraj GuhaThakurta, Jonathan Derry, John D Storey, Iliana Avila-Campillo, Mark J Kruger, Jason M Johnson, Carol A Rohl, Atila van Nas, Margarete Mehrabian, Thomas A Drake, Aldons J Lusis, Ryan C Smith, F. Peter Guengerich, Stephen C Strom, Erin Schuetz, Thomas H Rushmore, Roger Ulrich

**Affiliations:** 1 Rosetta Inpharmatics, Seattle, Washington, United States of America; 2 Department of Biostatistics, University of Washington, Seattle, Washington, United States of America; 3 Department of Genome Sciences, University of Washington, Seattle, Washington, United States of America; 4 Department of Microbiology, Molecular Genetics, and Immunology, University of California Los Angeles, Los Angeles, California, United States of America; 5 Department of Medicine, University of California Los Angeles, Los Angeles, California, United States of America; 6 Department of Human Genetics, University of California Los Angeles, Los Angeles, California, United States of America; 7 Department of Pathology and Laboratory Medicine, University of California Los Angeles, Los Angeles, California, United States of America; 8 Department of Biochemistry, Vanderbilt University School of Medicine, Nashville, Tennessee, United States of America; 9 Center of Molecular Toxicology, Vanderbilt University School of Medicine, Nashville, Tennessee, United States of America; 10 Department of Pathology, University of Pittsburgh, Pittsburgh, Pennsylvania, United States of America; 11 Department of Pharmaceutical Sciences, Saint Jude Children's Research Hospital, Memphis, Tennessee, United States of America; 12 Drug Metabolism, Merck and Company, West Point, Pennsylvania, United States of America; University of Michigan, United States of America

## Abstract

Genetic variants that are associated with common human diseases do not lead directly to disease, but instead act on intermediate, molecular phenotypes that in turn induce changes in higher-order disease traits. Therefore, identifying the molecular phenotypes that vary in response to changes in DNA and that also associate with changes in disease traits has the potential to provide the functional information required to not only identify and validate the susceptibility genes that are directly affected by changes in DNA, but also to understand the molecular networks in which such genes operate and how changes in these networks lead to changes in disease traits. Toward that end, we profiled more than 39,000 transcripts and we genotyped 782,476 unique single nucleotide polymorphisms (SNPs) in more than 400 human liver samples to characterize the genetic architecture of gene expression in the human liver, a metabolically active tissue that is important in a number of common human diseases, including obesity, diabetes, and atherosclerosis. This genome-wide association study of gene expression resulted in the detection of more than 6,000 associations between SNP genotypes and liver gene expression traits, where many of the corresponding genes identified have already been implicated in a number of human diseases. The utility of these data for elucidating the causes of common human diseases is demonstrated by integrating them with genotypic and expression data from other human and mouse populations. This provides much-needed functional support for the candidate susceptibility genes being identified at a growing number of genetic loci that have been identified as key drivers of disease from genome-wide association studies of disease. By using an integrative genomics approach, we highlight how the gene *RPS26* and not *ERBB3* is supported by our data as the most likely susceptibility gene for a novel type 1 diabetes locus recently identified in a large-scale, genome-wide association study. We also identify *SORT1* and *CELSR2* as candidate susceptibility genes for a locus recently associated with coronary artery disease and plasma low-density lipoprotein cholesterol levels in the process.

## Author Summary

Genome-wide association studies seek to identify regions of the genome in which changes in DNA in a given population are correlated with disease, drug response, or other phenotypes of interest. However, changes in DNA that associate with traits like common human diseases do not lead directly to disease, but instead act on intermediate, molecular phenotypes that in turn induce changes in the higher-order disease traits. Therefore, identifying molecular phenotypes that vary in response to changes in DNA that also associate with changes in disease traits can provide the functional information necessary to not only identify and validate the susceptibility genes directly affected by changes in DNA, but to understand as well the molecular networks in which such genes operate and how changes in these networks lead to changes in disease traits. To enable this type of approach we profiled the expression levels of 39,280 transcripts and genotyped 782,476 SNPs in 427 human liver samples, identifying thousands of DNA variants that strongly associated with liver gene expression. These relationships were then leveraged by integrating them with genotypic and expression data from other human and mouse populations, leading to the direct identification of candidate susceptibility genes corresponding to genetic loci identified as key drivers of disease. Our analysis is able to provide much needed functional support for these candidate susceptibility genes.

## Abstract 3

Identifying changes in DNA that associate with changes in gene expression in human tissues elucidates the genetic architecture of gene expression in human populations and enables the direct identification of functionally supported candidate susceptibility genes in genomic regions associated with disease.

## Introduction

Recent large-scale, genome-wide association studies have now delivered a number of novel findings across a diversity of diseases, including age-related macular degeneration [1–3], heart disease [4,5], host control of HIV-1 [6], type I and II diabetes [7,8], and obesity [9]. However, despite this astonishing rate of success, the major challenge still remains to not only confirm that the genes implicated in these studies are truly the genes conferring protection from or risk of disease, but to elucidate the functional roles that these implicated genes play with respect to disease. Most of the genetic association studies reporting novel, highly replicated associations to disease traits do not provide experimental data supporting the putative functional roles a given candidate susceptibility gene may play in disease onset or progression. Even in cases where susceptibility genes are well studied, with well known functions, nailing down how these genes confer disease susceptibility can linger for years, or even decades, as has been the case for genes like *ApoE*, an Alzheimer disease susceptibility gene identified more than 15 years ago [10].

Complex networks of molecular phenotypes—gene expression (mRNA, ncRNA, miRNA, and so on), protein expression, protein state, and metabolite levels—respond more proximally to DNA variations that lead to variations in disease-associated traits. These intermediate phenotypes respond to variations in DNA that in turn can induce changes in disease associated traits. Because a majority of single nucleotide polymorphisms (SNPs) detected as associated with disease traits from the recent wave of genome-wide association studies (GWASs) do not appear to affect protein sequence, it is likely that these SNPs either regulate gene activity at the transcript level directly or link to other DNA variations involved in this type of regulatory role. Therefore, to uncover the genetic determinants affecting expression in a metabolically active tissue that is relevant to the study of obesity, diabetes, atherosclerosis, and other common human diseases, we profiled 427 human liver samples on a comprehensive gene expression microarray targeting more than 39,000 transcripts, and we genotyped DNA from each of these samples at 782,476 unique SNPs. The relatively large sample size of this study and the large number of SNPs genotyped provided the means to assess the relationship between genetic variants and gene expression with more statistical power than many previous studies allowed [11–13]. A comprehensive analysis of the liver gene expression traits revealed that thousands of these traits are under the control of well-defined genetic loci, with many of the genes having already been implicated in a number of human diseases. Here we demonstrate directly how integrating genotypic and expression data in mouse and human can provide much-needed functional support for candidate susceptibility genes identified in a growing number of genetic loci that have been identified as key drivers of disease from GWASs. Specifically, we highlight how the gene *RPS26* and not *ERBB3* is most strongly supported by our data as a susceptibility gene for a novel type 1 diabetes (T1D) locus that was recently identified in a large-scale GWAS [14] and subsequently extensively replicated in a number of cohorts [15]. We also identify *SORT1* and *CELSR2* as candidate susceptibility genes for a locus recently associated with coronary artery disease [16] and plasma low-density lipoprotein (LDL)-cholesterol levels [17,18].

## Results

To characterize the genetic architecture of gene expression in human liver, we compiled a tissue-specific human liver cohort (HLC), which comprised 427 Caucasian subjects (Table S1). DNA and RNA were isolated from all liver tissue samples. Each RNA sample was profiled on a custom Agilent 44,000 feature microarray composed of 39,280 oligonucleotide probes targeting transcripts representing 34,266 known and predicted genes, including high-confidence, noncoding RNA sequences. Each DNA sample was genotyped on the Affymetrix 500K SNP and Illumina 650Y SNP genotyping arrays. Analysis was restricted to those SNPs that had a genotyping call rate greater than 75%, a minor allele frequency greater than 4%, and that did not deviate significantly from Hardy-Weinberg equilibrium in the HLC. A total of 310,744 and 557,240 SNPs met these criteria from the Affymetrix and Illumina sets, respectively, resulting in a set of 782,476 unique SNPs (85,508 SNPs were in the intersection), referred to here as the analysis SNP set.

### Genome-Wide Screen for Putative *cis*- and *trans*-Acting Expression Quantitative Trait Loci

To identify expression quantitative trait loci (eQTL) that have putative *cis* and *trans* [19] regulatory effects on the liver gene expression traits, we tested all expression traits for association with each of the SNPs in the analysis SNP set typed in the HLC. The strongest putative *cis* eQTL for a given expression trait was defined as the SNP most strongly associated with the expression trait over all of the SNPs typed within 1 megabase (Mb) of the transcription start or stop of the corresponding structural gene. The association *p*-values were adjusted to control for testing of multiple SNPs and expression traits using two different methods: (1) a highly conservative Bonferroni correction method to constrain the study-wise significance level, and (2) an empirical false discovery rate (FDR) method that constrains the overall rate of false positive events. For *cis* eQTL, we only test for associations to SNPs that are within 1 Mb of the annotated start or stop site of the corresponding structural gene. To achieve a study-wise significance level of 0.05, the Bonferroni adjusted *p*-value threshold was computed as 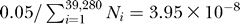 , where *N_i_* denotes the number of SNPs tested for trait *i*, over all 39,280 expression traits tested. At this threshold, 1,350 expression traits corresponding to 1,273 genes were identified.

The Bonferroni adjustment method can be conservative when there is dependence among the expression traits and among the SNP genotypes. Given that strong correlation structures exist among expression traits and among SNP genotypes in a givenlinkage disequilibrium (LD) block, the Bonferroni adjustment may be overly conservative. Therefore, we used an empirical FDR method based on permutations that accounts for the correlation structures among the expression traits and among the SNP genotypes. We constrained the empirically determined FDR to be less than 10% (see Methods). At this level, we identified 3,210 expression traits corresponding to 3,043 genes that were significantly associated with at least one SNP near the corresponding gene region (referred to here as a putative *cis* eQTL). The full list of association results are provided in Table S2. The magnitude of the effects ranged from SNPs that explained roughly 2% of the in vivo expression variation (*p* ∼ 0.003) to those that explained roughly 90% of the expression variation (*p* < 10^−65^).

Several recent studies have been published that examine the extent of genetic control in blood [20–22], brain [23], and adipose [21] gene expression via genetic association testing. In one of these studies [21], we performed the study on human blood and adipose tissues profiled on the same expression platform as the HLC, providing a straightforward way to compare the extent of *cis* eQTL overlap between blood, adipose, and liver tissues. In our characterization of blood and adipose tissue eQTL, there were 2,573 and 2,789 expression traits, respectively, represented on the microarray used to profile the HLC samples and that gave rise to *cis* eQTL. Of these, 752 blood and 881 adipose *cis* eQTL overlapped the set of 3,210 *cis* eQTL detected in the HLC. Therefore, in both adipose and blood, roughly 30% of the *cis* eQTL detected in these tissues were also detected in the HLC, confirming that there is significant, common genetic control between tissues. However, these overlaps also highlight that a majority of *cis* eQTL detected in one tissue may be specific to that tissue, potentially reflecting the genetic control of tissue-specific biological functions.

The significance of the *trans* eQTL in the HLC were also assessed by the Bonferroni method and by constraining the empirically determined FDR to be less than 10%. In the case of *trans* eQTL, all 782,476 SNPs were tested for association to each of the 39,280 expression traits. Therefore, the Bonferroni adjusted p-value threshold was computed as 0.05/(782,476 × 39,280) = 1.6 × 10^−12^. At this threshold, 242 expression traits corresponding to 236 genes were significantly associated with a SNP in *trans* (referred to here as a *trans* eQTL). On the other hand, by constraining the FDR to be less than 10%, 491 expression traits corresponding to 474 genes were identified as significantly associated with a SNP in *trans*. For the FDR-computed *cis* and *trans* eQTL signatures, the *trans* eQTL signature was only 15% the size of the *cis* eQTL signature, consistent with findings in other human genetics of gene expression studies [12,13]. The smaller *trans* eQTL signature likely reflects a lack of power to detect the small-to-moderate eQTL effects, given the sample size of this study in the context of testing 782,476 SNPs and profiling 39,280 expression traits. Other studies have noted strong heritability estimates for a majority of the expression traits that, when taken together with the small number of associations detected, suggests that expression in general is a complex trait under the control of many loci [21]. With the more stringent threshold required to constrain the FDR in searching for *trans* eQTL, the magnitude of the *trans* eSNP effects (mean *R* ^2^ = 0.19) was larger than the *cis* eSNP effects (mean *R* ^2^ = 0.14).

In this study we used both the Affymetrix and Illumina genotyping platforms, providing for increased power to detect *cis* and *trans* eQTL in the HLC compared to the detections achieved using the Affymetrix and Illumina sets independently [49]. Conditional on the sample size and FDR, the Illumina SNP set provided for roughly 15% more eQTLs than the Affymetrix SNP set, corresponding to a 15% increase in the relative power. This increase in power is primarily due to the higher genetic coverage of the Illumina SNP set compared to the Affymetrix SNP set. Further, given the ∼40,000 expression traits profiled in the HLC, we were able to estimate the genetic coverage of the Illumina and Affymetrix SNP sets on a cohort that is independent of the HapMap CEU (Utah residents with ancestry from Northern and Western Europe) subjects. Interestingly, we observed significantly lower genetic coverage (78%) than previously reported (90%) (electronic database: http://www.cidr.jhmi.edu/download/HumanHap650Y_info.pdf). Finally, in comparing whether more samples or more SNPs enhanced power most dramatically, we found that a modest increase in sample size (19%) had a more profound impact on the power to detect gene expression associations (a 21% increase in this case) than increasing the genetic coverage. These power and genetic coverage issues are fully detailed in a separate report [49].

The *cis* and *trans* eQTLs identified from the first pass analysis provide a significantly reduced set of SNPs on which to focus (∼3,700 versus 782,476). The set of SNPs associated with expression (eSNPs) can be considered a functionally validated set, given that the SNPs in this set have been found to associate with biologically relevant control of gene expression. In fact, many of the gene expression traits associated with eSNPs correspond to genes that have previously been associated with many different human diseases (Table S3). For example, *BRCA1*, a well-known susceptibility gene for breast cancer, and *CFH*, a susceptibility gene for age-related macular degeneration identified in one of the first published GWASs, are each strongly associated with an eSNP (*p* = 9.73 × 10^−17^ for *BRCA1* and *p* = 6.94 × 10^−22^ for *CFH*) that falls within 1Mb of the corresponding structural gene (Table S3). Genes associated with drug response are also represented in this set. For example, *VKORC1*, a gene recently associated with warfarin dosing [24], has liver gene expression values that are significantly associated with an eSNP near the 3′ end of the gene (*p* = 1.66 × 10^−23^).

To characterize further the effect that this set of eSNPs has on the liver transcriptional network, we re-analyzed the association results by restricting attention to this panel of SNPs. We again constrained the FDR to be less than 10% with respect to the eSNP set and identified an additional 3,053 expression traits, corresponding to 2,838 genes that were significantly associated with at least one of the eSNPs (Table S2). We assessed the significance of this new set of expression traits by randomly sampling five sets of SNPs from the full set of SNPs typed in the HLC, such that the size and minor allele frequency distribution matched that of the eSNP set. For each of the randomly selected SNP sets, we analyzed the associations between all expression traits and SNPs in this set. The maximum number of associations detected in any of the five sets at a 10% FDR was only 20, and the mean detection rate over all sets was 12. This demonstrates well the biological utility of the eSNP set, given that this set is significantly enriched for SNPs that associate with expression traits, beyond the initial set of expression traits that defined the eSNP set, compared to comparable sets of randomly selected SNPs. A number of eQTL hot spots emerged as well in this full set of expression traits, where a given locus was identified as a hot spot if greater than 20 expression traits linked to a single eSNP at the locus. The significance of these hot spots was assessed by permuting the genotypes and examining the distribution of associations in the permuted sets. In each permutation set, we identified the maximum number of traits associated with a single marker over all markers. The mean of the maximum counts over 10 permutation sets was only 12, compared to a maximum of 283 in the observed data (Table S2).

### Integrating Genetic and Network Data across Species to Inform GWA Discoveries

Identifying candidate susceptibility genes in regions associated with disease using the proximity of the candidate genes to SNPs in that region may be misleading a lot of the time. For example, from Table S2, for the 3,210 expression traits giving rise to *cis* eQTLs, only 627 of the corresponding *cis* eSNPs are located within the corresponding gene region, whereas 1,282 are located downstream of the 3′ untranslated region (UTR) and 1,301 are located upstream of the 5′ UTR. Further, of the *cis* eSNPs located up- and downstream of the corresponding genes, 490 and 526, respectively, are >100 kb away. That is, greater than 30% of all *cis* eSNPs fall greater than 100 kb away from the transcription start and stop sites of the corresponding gene. Therefore, at least for expression traits, the nearest SNP rule for inferring genes given an association finding would result in an unacceptably high miss-call rate. Genes with expression values that are strongly associated with variations in DNA provide a different path to elucidate the gene or genes and their respective functions underlying genetic loci associated with disease in a more objective fashion.

#### Identifying candidate susceptibility genes for T1D.

In one of the largest GWASs carried out to date, the Wellcome Trust Case Control Consortium (WTCCC) studied 14,000 cases and 3,000 shared controls with respect to seven common diseases [14]. T1D was one of the key disease focuses of this study, with a number of replications reported simultaneously in a separate follow-up study [15]. In addition, a number of T1D susceptibility genes identified prior to the WTCCC study have been identified and more thoroughly replicated, including the HLA class II genes *INS*, *CD25*, *CTLA4*, *PTPN22*, and *IFIH1*. Given that the SNPs genotyped in the WTCCC study were also genotyped in the HLC, we examined the extent to which the T1D SNPs identified in the WTCCC study were associated with the expression traits corresponding to the genes implicated in the study.

Table 1 highlights nine genes previously identified as T1D susceptibility genes or inferred as T1D susceptibility genes from the WTCCC study. The expression levels for five of these genes (*CTLA4*, *HLA*-*DRB1*, *IL2RA*, *LONRF2*, and *CHST10*) in the HLC were associated with the corresponding T1D-associated SNP. For *IL2RA* and the four other genes (*AFF3*, *ADAD1*, *PTPN2*, and *IL2*), the expression levels were associated with other SNPs in the region of the T1D-associated SNPs*.* We also examined whether other genes in the vicinity of the T1D-associated SNPs had expression levels that were also associated with these SNPs. An additional four genes highlighted in Table 1 (*RPS26*, *CLECL1*, *IGF2AS*, and *Hct1837134*) were identified in this way, in addition to two HLA class II genes highlighted in Table 2 (*HLA*-*DQB1* and *HLA*-*DQA2*). Given the role that HLA class II genes are known to play in T1D, we also examined the 14 HLA class II gene expression traits represented on the array used in this study, and we found that 11 of them gave rise to significant genetic associations (Table 2). Some of the associations were striking and highlight additional SNPs that may be of interest in genetic disease association studies. For example, greater than 50% of the *HLA-DRB5* expression variation observed in the HLC could be explained by a single *cis* eSNP (rs9271366).

**Table 1:**
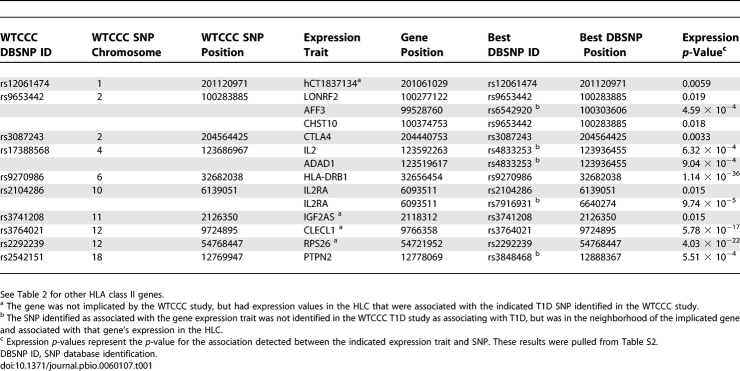
Expression Traits Corresponding to Genes Implicated in the T1D WTCCC Study [14,15] or Close to Genes Associated with Either SNPs That Were Associated with T1D in the WTCCC Study or with SNPs Close the T1D-Associated SNPs

**Table 2:**
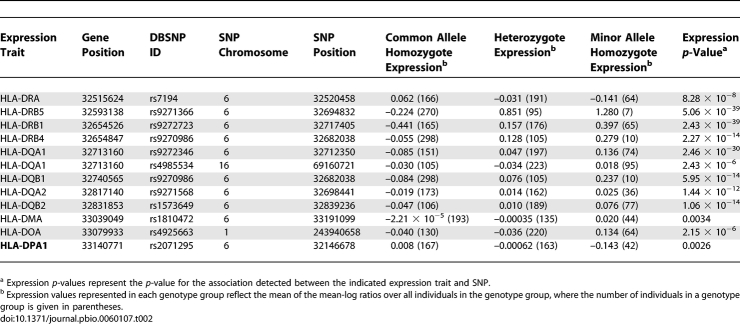
Significant Associations Detected in the HLC for 11 of the 14 HLA Class II Gene Expression Traits Represented on the Microarray Used in This Study

The absence of an association between a T1D-associated SNP and the HLC expression values corresponding to a candidate susceptibility gene for that SNP cannot be taken as strong evidence against the gene's candidacy as a susceptibility gene. The underlying causal change in DNA may not affect expression levels of the gene in question, or the variation in expression may be specific to a given tissue not profiled or to conditions not reflected in the HLC. However, strong associations between T1D-associated SNPs and expression levels of genes near the SNP provide direct functional support for a gene's involvement in disease susceptibility. For example, rs3764021 was identified as a T1D susceptibility locus in the WTCCC study and then extensively replicated [15]. *CLEC2D* was inferred as the most likely susceptibility gene at this locus. However, *CLEC2D* expression in the HLC data was not associated with this SNP; but a flanking gene, *CLECL1* was significantly associated (*p* = 5.78 × 10^−17^; Table 1). Given that *CLEC2D* and *CLECL1* are in the same gene family, the strong association between the T1D SNP and *CLECL1* expression data suggest that *CLECL1* may be a better candidate susceptibility gene to examine.

In cases where disease-associated traits and expression traits are scored in the same cohort, there is the potential to directly infer causal relationships between genes and disease [25]. However, even without disease trait data in tissue-specific cohorts like the HLC, an integrative genomics approach can be used to identify the most likely candidate susceptibility gene for a given locus. For example, one of the more novel regions associated with T1D from the WTCCC study was Chromosomes 12q13 (rs2292239). *ERBB3*, a receptor tyrosine-protein kinase with a presumed role in immune signaling, was identified as the most plausible susceptibility gene at this locus. While *ERRB3* expression in the HLC was not associated with this SNP, the expression of a flanking gene, *RPS26*, was significantly associated with this SNP (*p* = 4.03 × 10^−22^; Table 1). In fact, 40% of the in vivo expression variation for *RPS26* in the HLC was explained by this single T1D associated SNP, and this SNP was the most strongly associated with *RPS26* expression out of the greater than 800,000 SNPs genotyped in the HLC, .

The association to *RPS26* expression suggests that this gene warrants further study in the context of T1D. However, these data on their own are still far from conclusive, given there may be DNA variants that affect RPS26 expression independently of T1D, but where these variants are in strong LD with the DNA variants explaining the T1D susceptibility. Therefore, to further explore the role *RPS26* and *ERBB3* may play in T1D, we examined the expression data for these genes in an expression atlas for human, monkey, and mouse, where for each species, between 45 and 60 tissue samples were profiled [26,27]. Although both genes are expressed in mouse, monkey, and human tissues, the expression of *RPS26* is >1–2 log units higher in the pancreas and islets of Langerhan compared to *ERBB3* (Figure S1), with *ERBB3* observed as lowly expressed in islets as measured in the mouse body atlas. Given the central role that pancreas and islets play in T1D, these results further suggest *RPS26* as a candidate susceptibility gene for T1D.

**Figure 1:**
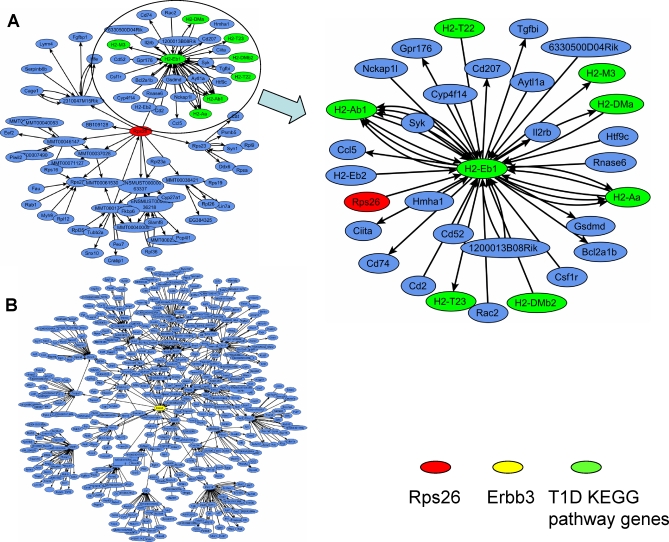
Local Networks for *Rps26* and *Erbb3* Derived from Causal, Probabilistic Whole-Gene Networks Constructed from the Liver, Adipose, Muscle, and Brain Gene Expression Data Generated from the BXH/wt and BXC Mouse Crosses (A) The *Rps26* subnetwork includes a number of known T1D associated genes (green nodes), and *RPS26* in this subnetwork is directly linked to *H2-Eb1*, a mouse ortholog of *HLA-DRB1*, a previously identified T1D susceptibility gene that is also strongly associated with a *cis* eSNP in the HLC (Table 2). The known T1D genes annotated by the Gene Ontology are significantly enriched in this subnetwork (Table 3). (B) The *Erbb3* subnetwork is not associated with any pathways known or predicted to be involved in T1D.

What the genetic association and atlas data lack is a more refined context within which to assess the functional role a given gene plays in a system. We have previously described a method to reconstruct probabilistic, causal networks by integrating genetic and gene expression data [25,28–30]. Examining candidate susceptibility genes in the context of these networks can provide insights into the pathways in which they operate. We constructed whole-gene networks from three F2 intercross populations constructed from the B6, C3H, and CAST strains (see Methods for details). Liver and adipose expression data were generated from these populations and integrated with the genotypic data also generated in these populations to reconstruct the networks as previously described [28,30]. We then examined *RPS26* and *ERBB3* in the context of these networks (Figure 1).

Figure 1A highlights how *RPS26* is directly connected to a number of known T1D genes. For example, *RPS26* is directly connected to a mouse ortholog of *HLA*-*DRB1*, a gene previously associated with T1D and highlighted in this present study as having liver expression values that are strongly associated with a highly replicated T1D SNP (Table 1). In fact, the genes comprising the local network structure around *RPS26* are enriched for genes annotated as T1D genes, in addition to being enriched for genes operating in a number of pathways commonly associated with T1D (Table 3). On the other hand, whereas *ERBB3* also resided in the context of a well defined subnetwork (Figure 1B), the genes comprising this subnetwork were not enriched for any T1D associated pathways.

**Table 3:**
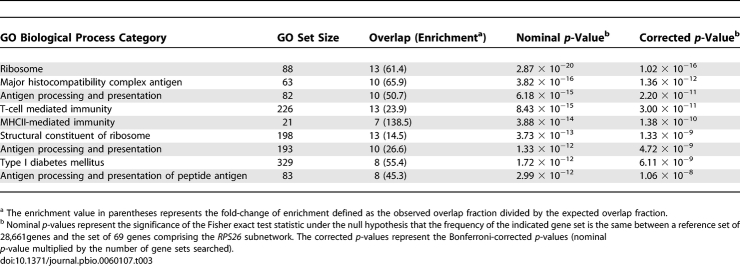
GO Biological Process Categories Enriched in the *RPS26* Subnetwork Depicted in Figure 1A

#### Identifying candidate susceptibility genes for coronary artery disease and LDL cholesterol levels.

**Figure 2:**
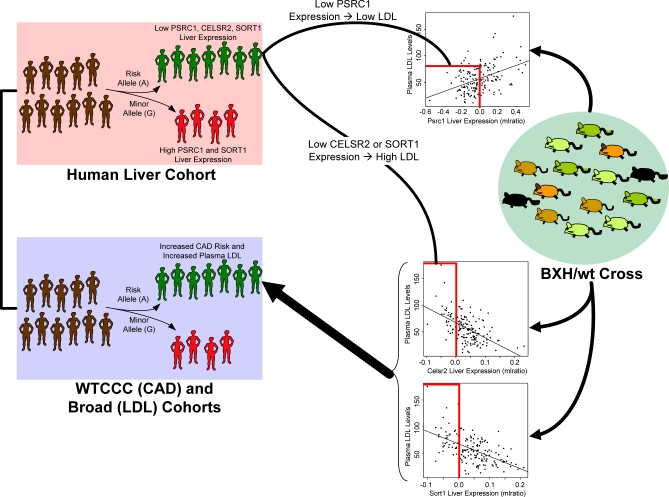
*PSRC1*, *CELSR2*, and *SORT1* Liver Expression Is Associated with a CAD Risk Allele and Plasma LDL Cholesterol Levels The CAD risk allele for SNP rs599839 was established in a previous WTCCC study [16] (lilac panel). In the HLC, this same SNP is strongly associated with *PSRC1*, *CELSR2*, and *SORT1* expression, with the CAD risk allele associated with lower relative expression (pink panel). In the BXH/wt cross designed to study metabolic traits that increase cardiovascular risk (green panel), all three of these expression traits were strongly correlated with plasma LDL cholesterol levels, a major CAD risk factor (scatter plots associated with the green panel). Given the association of these genes to plasma LDL-cholesterol levels, we examined whether rs599839 was associated with LDL cholesterol in a previously published GWAS [35] and found this SNP was significantly associated with LDL cholesterol levels, where the CAD risk allele was associated with higher LDL cholesterol levels in this cohort. Lower levels of *CELSR2* and *SORT1* expression were associated with the risk allele in humans, and with higher LDL cholesterol levels in mouse, making them ideal candidate susceptibility genes for the CAD and LDL cholesterol associations to this locus. On the other hand, lower levels of *PSRC1* expression were associated with the risk allele in humans, but with lower LDL cholesterol levels in mouse, suggesting that *PSRC1* is not the gene increasing CAD risk, but instead may be acting to protect against it.

Another GWAS involving the WTCCC resulted in the identification of seven loci associated with coronary artery disease (CAD) [16]. The seven top-hitting SNPs associated with CAD at each of the seven loci in this study were represented on the Affymetrix 500K array. Therefore, we examined the HLC data to identify expression traits that were significantly associated with any of the seven CAD-associated SNPs. Given the roughly 40,000 expression traits examined at each of the seven SNPs (280,000 tests in all), we set a nominal *p*-value threshold of 0.05/280,000 = 1.79 × 10^−7^ for significance. Only one of the seven SNPs identified in the WTCCC CAD study, rs599839 on Chromosome 1p13.3, was significantly associated with any of the HLC expression traits (Figure 2). Four different expression traits were identified as significantly associated with rs599839 (Table 4). One of the four expression traits corresponded to a gene, *PSRC1*, that had been identified as a candidate susceptibility gene in the WTCCC CAD study [16].

**Table 4:**
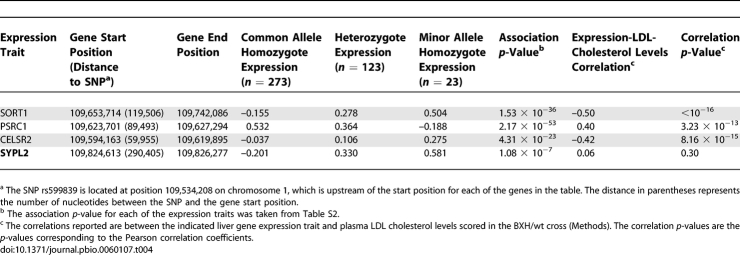
Significant Associations Detected between Liver Expression Traits in the HLC and the CAD-Associated SNP, rs599839, on Chromosome 1p13.3

To further characterize the association of these four expression traits with CAD-associated traits, we examined the activity of these genes in the BXH/wt cross (see Methods for details), a cross designed specifically to study metabolic traits that increase risk of cardiovascular disease. The liver expression levels of *Psrc1*, *Sort1*, and *Celsr2*, but not *Sypl2*, in the BXH/wt cross were significantly associated with plasma LDL cholesterol levels (Table 4), a major CAD risk factor. However, while *Psrc1* expression levels were positively correlated with plasma LDL cholesterol levels, *Sort1* and *Celsr2* expression levels were negatively correlated. In addition, for liver expression traits in the BXH/wt cross significantly correlated with these three genes, the Sort1 and Celsr2 correlation signatures were most significantly enriched for the GO Biological Process category “cell surface receptor linked signal transduction” (1.4-fold enrichment, *p* = 1.91 × 10^−5^, and 1.6-fold enrichment, *p* = 4.57 × 10^−10^, for *Sort1* and *Celsr2*, respectively), while the *Psrc1* correlation signature was most enriched for the “cell cycle” category (3-fold enrichment, *p* = 0.00044), suggesting that *Sort1* and *Celsr2* may be involved in similar biological processes that are distinct from processes involving *Psrc1*.

**Figure 3:**
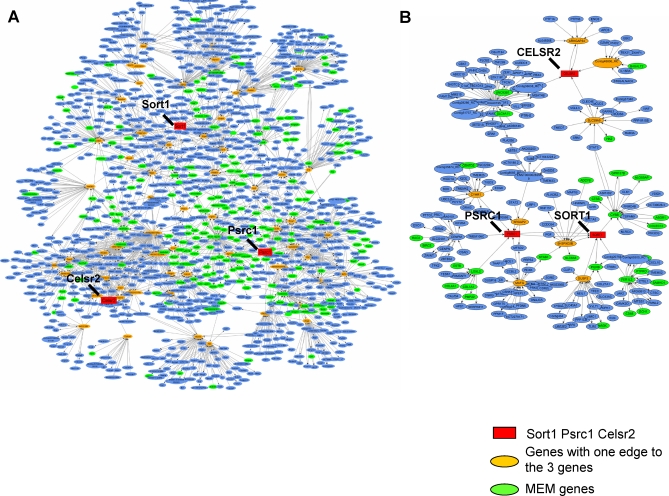
Local Networks for *PSRC1*, *CELSR2*, and *SORT1* Derived from Causal, Probabilistic Whole-Gene Networks in Mouse and Human (A) Mouse network for *Psrc1*, *Celsr2*, and *Sort1* derived from the liver, adipose, muscle, and brain gene expression data generated from the BXH/wt and BXC mouse crosses. (B) Human network for *PSRC1*, *CELSR2*, and *SORT1* derived from the HLC and from a previously published adipose and blood tissue cohort [21].

To further elucidate the involvement of these genes in metabolic phenotypes associated with CAD, we examined *Psrc1*, *Celsr2*, and *Sort1* in the context of the probabilistic, causal network constructed as described above for the *Erbb3*/*Rps26* example. All three genes not only fell in the same subnetwork, they were all directly connected to the same gene, *2010200O16Rik*, demonstrating that these genes are tightly co-regulated, possibly driven by common regulatory factors (Figure 3A). This same subnetwork also included genes like *Tgfbr2*, *Pparg*, *Lpl*, *Ppm1l*, and *Alox5ap*, all of which have been previously identified and validated as being associated with traits related to obesity, diabetes, cholesterol levels, and cardiovascular disease [25,31–33]. More generally, *Psrc1* and *Sort1* participate in a previously defined macrophage-enriched metabolic (MEM) subnetwork validated as causal for obesity-, diabetes-, and atherosclerosis-related traits [34]. In fact, the subnetwork depicted in Figure 3A is composed of 1,346 genes, with 226 of these genes overlapping the set of 1,406 genes composing the MEM subnetwork (82 would have been expected by chance). This 2.76-fold enrichment in this case is highly significant, with a Fisher exact test *p* = 8.20 × 10^−47^.

To establish whether *PSRC1*, *CELSR2*, and *SORT1* are closely connected in human transcriptional networks as they are in mouse, we constructed a probabilistic, causal network from the HLC and from a previously published adipose and blood tissue cohort [21], using previously described methods [25,28–30]. As depicted in Figure 3B, *PSRC1*, *CELSR2*, and *SORT1* fall in the same subnetwork and are closely connected, as in the mouse network. In addition, the genes comprising this human subnetwork are enriched for genes that fall in the mouse network depicted in Figure 3A (Fisher exact test *p* = 1.78 × 10^−8^). Further, the human subnetwork is also enriched for genes falling in the MEM module (Fisher exact test *p* = 5.03 × 10^−8^), confirming the association to metabolic phenotypes detected in the mouse network. These data combined suggest that *PSRC1*, *CELSR2*, and *SORT1* operate in a conserved subnetwork causally associated with cholesterol levels, obesity, diabetes and atherosclerosis.

Given the strong association between plasma LDL cholesterol levels and the expression of *Psrc1*, *Sort1*, and *Celsr2* expression in the BXH/wt cross, we examined a recent GWAS available in the public domain in which LDL cholesterol levels were monitored [35]. A significant association was detected between rs599839 genotypes and LDL cholesterol levels in this human cohort (*p* = 9.0 × 10^−8^)[35]. Interestingly, the common allele for rs599839 was associated with higher LDL cholesterol levels [35], consistent with the association of this allele with increased CAD risk. Low *SORT1*, *CELSR2*, and *PSRC1* expression levels in the HLC are also associated with the rs599839 common allele. However, given low *Sort1* and *Celsr2* expression levels in the BXH/wt cross are associated with increased LDL cholesterol levels (whereas low *Psrc1* expression levels are associated with low LDL cholesterol levels), *SORT1* and *CELSR2* are the most logical candidate susceptibility gene in the 1p13.3 locus (Figure 2), although direct experimental manipulation of these two genes would be required to provide more direct functional support that these genes are involved in modulating LDL cholesterol levels. The association of this locus with LDL cholesterol levels as well as liver expression levels of *SORT1*, *CELSR2*, and *PSRC1* were recently reported in multiple independent studies [17,18].

## Discussion

Previous studies on the genetics of gene expression in humans have focused primarily on lymphoblastoid cell lines or other blood-derived samples [13,14,17]. We have provided a large-scale assessment of the genetics of gene expression in human liver, a metabolically active tissue that is critical to a number of core biological processes and that plays a role in a number of common human diseases. After profiling 427 human liver samples on a comprehensive gene expression microarray and genotyping the DNA from these samples at greater than one million SNPs, we identified a significant genetic signature underlying the expression of more than 6,000 genes, with many of these genes already implicated as causal for a number of different diseases, including heart disease, breast cancer, inflammatory bowel disease, age-related macular degeneration, schizophrenia, and Alzheimer disease. This set of data highlights the utility of monitoring molecular phenotypes that underlie the higher order clinical states of a system.

Whereas the eQTL data in the human liver cohort is valuable in its own right, when integrated with other GWAS data and with genetics of gene expression and clinical data in segregating mouse populations, there is the potential to directly identify experimentally supported candidate susceptibility genes for disease. We demonstrated directly how genetics of gene expression data can complement multiple GWAS datasets by highlighting *SORT1* and *CELSR2* as candidate susceptibility genes for CAD and LDL cholesterol levels at a recently identified locus associated with CAD [16]. In this instance, the association to LDL cholesterol levels is novel and based on publicly available GWAS data and a mouse cross designed specifically to study lipid and other metabolic syndrome traits.

In addition to the CAD locus, we highlighted *RPS26* as a candidate susceptibility gene for T1D from a novel, highly replicated T1D locus on Chromosome 12q13, which was identified in a separate GWAS [15]. Not only was the expression of this gene in the HLC strongly associated with the T1D SNP at this locus, but it was observed to operate in a part of the molecular network that is significantly enriched for genes associated with T1D (like *HLA*-*DRB1*), whereas the gene inferred as the most likely susceptibility gene at that locus (*ERBB3*) [15] was not supported by any of our experimental data. Recent studies have demonstrated that ribosomal proteins may be involved in auto-immune diseases like systemic lupus erythematosus [36]. In addition, recent work has demonstrated a connection between endoplasmic reticulum (ER) stress in the cytoplasm and diabetes, where protein unfolding in response to ER stress is hypothesized to disrupt processes associated with diabetes [37]. Given *RPS26*'s protein translation role as part of the ribosomal complex on the ER, its association to T1D is particularly intriguing. The unfolded protein response has also been linked to inflammation and oxidative stress [38], hence the putative connection between *RPS26* and an auto-immune disease like T1D is worthy of further consideration. Cells with high secretory capacity like pancreatic beta cells are also more likely to be susceptible to ER stress, making the link between *RPS26* and T1D even more plausible. In fact, previous work has indicated higher ER stress levels in T1D patients [39].

It is important to note that a lack of association between expression traits in the HLC and disease-associated SNPs is not a valid filter for excluding a gene as a candidate disease susceptibility gene, given that variation in a gene leading to disease may affect protein function and not expression, or it may affect expression in a different tissue or under different environmental conditions. However, the approach of analyzing the genetics of gene expression in human populations does provide a more objective view into the functioning of genes in a given disease-associated region. This view has the potential to lead to higher confidence candidates in the absence of direct functional support for any one gene, which is typically the case in GWASs where the SNPs identified have no known functional role. Given the potential that genetics of gene expression studies have to affect our understanding of common human diseases, generating even larger-scale molecular profiling datasets in segregating populations may provide a path to more rapidly elucidating not only the genetic basis of disease, but the impact the genetic basis of disease has on molecular networks that in turn induce variations in disease associated traits.

## Materials and Methods

### HLC and tissue collection.

The HLC was assembled from a total of 780 liver samples (1–2 g) that were acquired from Caucasian individuals from three independent liver collections at tissue resource centers at Vanderbilt University, the University of Pittsburgh, and Merck Research Laboratories (Table S1). The Vanderbilt samples (*n* = 504) included both postmortem tissue and surgical resections from organ donors and were obtained from the Nashville Regional Organ Procurement Agency (Nashville, Tennessee), the National Disease Research Interchange (Philadelphia, Pennsylvania), and the Cooperative Human Tissue Network (University of Pennsylvania, Ohio State University, and University of Alabama at Birmingham). The Pittsburgh samples were normal postmortem human liver and were obtained through the Liver Tissue Procurement and Distribution System (Dr. Stephen Strom, University of Pittsburgh, Pittsburgh, Pennsylvania). The University of Pittsburgh samples (*n* = 211) were all postmortem, as were the Merck samples (*n* = 65), which collected by the Drug Metabolism Department and reported previously [40].

All samples were stored frozen at −80 °C from collection until processing for RNA and DNA; some samples had been stored for over a decade before being processed for this study. Demographic data varied across centers for these samples and were missing in many cases. In cases where age, sex, or ethnicity data were not available in the patient records, we imputed it from the gene expression and/or genotype data (see below). Of the 780 samples collected, high-quality DNA was isolated on 548 samples, and 517 of these were successfully genotyped on the Affymetrix genotyping platform (see Methods below). Of the 517 successfully genotyped samples, high-quality RNA was isolated and successfully profiled on 427 samples. This set of 427 genotyped and expression profiled samples comprised the HLC. Table S1 gives a summary of the demographics and other annotations on the 427 individuals that were successfully genotyped and expression profiled. All counts and descriptive statistics include the imputed data. All samples and patient data were handled in accordance with the policies and procedures of the participating organizations.

### Mouse crosses and tissue collection.

C57BL/6J (B6) mice were intercrossed with C3H/HeJ (C3H) mice to generate 321 F2 progeny (161 females, 160 males) for the BXH wild type (BXH/wt). C57BL/6J (B6) mice were intercrossed with Castaneus (CAST) mice to generate 442 F2 progeny (276 females, 166 males) for the BXC cross. All mice were maintained on a 12 h light–12 h dark cycle and fed ad libitum. BXH mice were fed Purina Chow (Ralston-Purina) containing 4% fat until 8 wk of age. From that time until the mice were killed at 20 wk, mice were fed a western diet (Teklad 88137, Harlan Teklad) containing 42% fat and 0.15% cholesterol. BXC mice were fed Purina Chow until 10 wk of age, and then fed western diet (Teklad 88137, Harlan Teklad) for the subsequent 8 wk. Mice were fasted overnight before they were killed. Their livers were collected, flash frozen in liquid nitrogen, and stored in −80 °C prior to RNA isolation.

The BXH cross on an ApoE null background (BXH/apoE) was previously described [41]. Briefly, C57BL/6J ApoE null (B6.ApoE^–/–^) were purchased from Jackson Laboratory. C3H/HeJ ApoE null (C3H.Apo E^–/–^) were generated by backcrossing B6.ApoE^–/–^ to C3H for ten generations. F1 mice were generated from reciprocal intercrossing between B6.ApoE^–/–^ and C3H.ApoE^–/–^, and F2 mice were subsequently bred by intercrossing F1 mice. A total of 334 (169 female, 165 male) were bred, and all were fed Purina Chow containing 4% fat until 8 wk of age, and then transferred to western diet containing 42% fat and 0.15% cholesterol for 16 wk. Mice were killed at 24 wk, and liver, white adipose tissue, and whole brains were immediately collected and flash-frozen in liquid nitrogen.

All procedures of housing and treatment of animals were performed in accordance with Institutional Animal Care and Use Committee regulations.

### Microarray design, RNA sample preparation, hybridization, and expression analysis.

*Array design and preparation of labeled cDNA and hybridizations to microarrays for the human liver cohort.* RNA preparation and array hybridizations were performed at Rosetta Inpharmatics. The custom ink-jet microarrays used in this study were manufactured by Agilent Technologies and consisted of 4,720 control probes and 39,280 noncontrol oligonucleotides extracted from mouse Unigene clusters and combined with RefSeq sequences and RIKEN full-length cDNA clones (Table S4).

Liver samples extracted from the 427 Caucasian individuals were homogenized, and total RNA extracted using TRIzol reagent (Invitrogen) according to manufacturer's protocol. Three micrograms of total RNA was reverse transcribed and labeled with either Cy3 or Cy5 fluorochrome. Purified Cy3 or Cy5 complementary RNA was hybridized to at least two single microarrays with fluor reversal for 24 h in a hybridization chamber, washed, and scanned using a laser confocal scanner. Arrays were quantified on the basis of spot intensity relative to background, adjusted for experimental variation between arrays using average intensity over multiple channels, and fitted to an error model to determine significance (type I error), as previously described [42]. Gene expression is reported as the mean-log ratio relative to the pool derived from 192 liver samples selected for sex balance from the Vanderbilt and Pittsburgh samples, because the RNA from the Merck samples had been amplified at an earlier date. The error model used to assess whether a given gene is significantly differentially expressed in a single sample relative to a pool composed of a randomly selected subset of samples has been extensively described and tested in a number of publications [42–44].

The age, sex, race, center, alcohol use, drug use, and steatosis variables presented in Table S1 were tested for association to the gene expression traits. Only age, sex, race, and center were significantly associated with the expression traits beyond what would be expected by chance. As a result, all gene expression traits were adjusted for these covariates. The lack of association between the expression traits and alcohol use, drug use, and steatosis was somewhat surprising, but may be due to the sparseness of these data, resulting in a lack of power to detect significant associations.

*Array design and preparation of labeled cDNA and hybridizations to microarrays for the mouse liver and adipose tissue samples.* RNA preparation and array hybridizations were again performed at Rosetta Inpharmatics. The custom ink-jet microarrays used in the BXH/wt, BXH/apoE, and BXC crosses were manufactured by Agilent Technologies. The array used for the BXH/apoE and BXH/wt samples consisted of 2,186 control probes and 23,574 noncontrol oligonucleotides extracted from mouse Unigene clusters and combined with RefSeq sequences and RIKEN full-length cDNA clones (Table S5). The array used for the BXC cross consisted of 39,280 noncontrol oligonuceotides again extracted from the mouse Unigene clusters and combined with RefSeq sequences and RIKEN full-length cDNA clones (Table S6).

Mouse adipose and liver tissues from all of the crosses were homogenized, and total RNA extracted using Trizol reagent (Invitrogen) according to manufacturer's protocol. Three micrograms of total RNA was reverse transcribed and labeled with either Cy3 or Cy5 fluorochrome. Labeled complementary RNA (cRNA) from each F2 animal was hybridized against a cross-specific pool of labeled cRNAs constructed from equal aliquots of RNA from 150 F2 animals and parental mouse strains for each of the three tissues for each cross. The hybridizations for the BXH/apoE cross were performed in fluor reversal for 24 h in a hybridization chamber, washed, and scanned using a confocal laser scanner. The hybridizations for the BXH/wt and BXC crosses were performed to single arrays (individuals F2 samples labeled with Cy5 and reference pools labeled with Cy3 fluorochromes) for 24 h in a hybridization chamber, washed, and again scanned using a confocal laser scanner. Arrays were quantified on the basis of spot intensity relative to background, adjusted for experimental variation between arrays using average intensity over multiple channels, and fitted to a previously described error model to determine significance (type I error) [42]. Gene expression measures are reported as the ratio of the mean log_10_ intensity (mlratio).

### DNA processing.

*DNA isolation*. DNA isolation was performed at Rosetta Inpharmatics. DNeasy tissue kits from QIAGEN were used to carry out all DNA extractions. For each liver sample, 20–30 mg of liver was placed in a 1.5-ml microcentrifuge tube along with 80 μl buffer ATL and 20 μl proteinase K. The contents of each tube were then mixed thoroughly by vortexing, followed by incubation at 55 °C until the tissue was completely lysed. Transcriptionally active tissues such as liver and kidney contain high levels of RNA, which will co-purify with genomic DNA. Because RNA-free genomic DNA was required for processing, 4 μl RNase A (100 mg/ml) was added and mixed by vortexing, followed by incubation for 2 min at room temperature before continuing. Samples were then vortexed and 200 μl buffer AL was added to the sample and mixed thoroughly. After 10 min incubation at 70 °C, 200 μl ethanol (96%–100%) was then added and mixed again. The mixture was placed into the DNeasy Mini column and centrifuged at 6,000*g* (8,000 rpm) for 1 min. The DNeasy Mini spin column was then placed in a new 2-ml collection tube, and 500 μl buffer AW1 was added, followed by placement in a centrifuge for 1 min at 6,000*g* (8,000 rpm). The DNeasy Mini spin column was then placed in a new 2-ml collection tube again, and 500 μl buffer AW2 was added and centrifuged for 3 min at 20,000*g* (14,000 rpm) to dry the DNeasy membrane. Then the DNeasy Mini spin column was placed in a clean 1.5-ml or 2-ml microcentrifuge tube and 200 μl buffer AE was pipetted directly onto the DNeasy membrane. This was incubated at room temperature for 1 min and then centrifuged for 1 min at 6,000*g* (8,000 rpm) to elute. Two 200-μl elutions were performed followed by ethanol/sodium acetate precipitation and resuspension of the resultant pellet with TE buffer.

*Genotyping data from the Affymetrix 500K panel.* SNP genotyping was performed with the commercial release of the Affymetrix 500K genotyping array. The genotyping was carried out at the Perlegen genotyping facility in Mountain View, California. Genotyping was attempted on 548 samples. 18 samples were unable to be genotyped because of poor DNA quality, and an additional 13 samples were removed after genotyping because their overall call rate did not exceed the 90% cutoff we required. We then applied SNP-wise quality checks on the 517 samples that were successfully genotyped. The Affymetrix 500K array consisted of 500,568 SNPs in total, 429,545 SNPs provided quality data from the genotyping assay, and we rejected those SNPs with a call rate < 75%, resulting in a final panel of 393,494 SNPs. We further filtered out SNPs with minor allele frequencies < 4% (81,646 SNPs) or SNPs that deviated from Hardy-Weinberg equilibrium (*p* < 10^−4^; 1,104 SNPs). The resulting set of 310,744 SNPs were used to carry out tests for association to the liver gene expression traits in the HLC.

*Genotyping data from the lllumina 650Y panel.* SNP genotyping was performed on the same set of samples that were genotyped on the Affymetrix 500K panel using the Sentrix humanHap650Y genotyping beadchip from Illumina. The genotyping was carried out at the Illumina genotyping facility in La Jolla, California. This chip consists of 655,352 tag SNP markers derived from the International HapMap Project (http://www.hapmap.org) on a single BeadChip, with ∼100,000 Yoruba-specific tag SNPs to provide more comprehensive coverage in African and African-American populations. Genotyping was attempted on 517 samples. A total of 497 samples were genotyped successfully, and 654,069 SNP assays genotyped successfully. The same genotype quality control measures applied to the Affymetrix 500K dataset were applied to Illumina HumanHap 650Y dataset to determine the analysis set. The sample set for analysis (*n* = 397) was restricted to those identified or imputed as Caucasian. Of the 397 samples we attempted to genotype, 13 failed the Illumina genotyping assay (overall call rate < 75%), resulting in a set of 384 genotyped samples carried forward for the expression analysis. In total, 652,648 SNPs were called, with only two SNPs rejected because the call rate was <75%. We then sequentially removed 94,915 SNPs with MAF <4% and 491 SNPs that deviated from the Hardy-Weinberg equilibrium (*p* < 10^−4^). The resulting set of 557,240 SNPs was used to carry out tests for association to the liver gene expression traits in the HLC.

A total of 85,508 SNPs were represented in both the Illumina and Affymetrix SNP sets. Therefore, there were 782,476 unique SNPs successfully genotyped in the HLC such that the call rate was greater than 75%, the MAF >4%, and there was not significant deviation from Hardy-Weinberg equilibrium at the 0.0001 significance level. The sample set for analysis was restricted to the 427 HLC samples that had both genotype and gene expression data available, passed the criteria outlined above and those that were identified as Caucasian, or imputed to be Caucasian when data was missing (see below).

### Data preprocessing.

*Sex confirmation.* Sex identifiers were available for most of the liver samples obtained from the three study centers. We independently confirmed the sex of each individual providing a liver sample by two methods. First, we looked for expression of Y-specific genes in the liver gene expression based on three probes representing three distinct transcripts. Second, we scored heterozygosity of X-chromosome markers. We excluded any individual for which there was a discrepancy in any of the three measures of sex in order to ensure a coherent data set for analysis and that we had excluded as many potential cases of annotation or sample-handling errors as possible. For samples where sex was not noted in the records, we imputed the sex call if both the genotype and gene-expression data were concordant.

*Ethnicity*. Ethnicities were confirmed or imputed using STRUCTURE [45]. A panel of 106 autosomal markers was randomly selected from around the genome to be unlinked and ancestry informative. Markers were selected from the HapMap data [46] that were present on the Affy 500K panel such that the minor allele frequency was >0.05 and the absolute allele frequency difference in the Caucasians and African Americans ∼0.5, with average minor allele frequency 0.5 (standard deviation = 12). Several *K* were tested (*K* = 1–6) with burn-in 100,000 and 100,000 reps of MCMC before any information was collected. In all cases, the greatest support was for *K* = 2. Admixture was detected for some individuals in some runs and some individuals were reclassified. For those unknown and reclassified, population reassignment was made if the probability of group membership was >0.9 for that individual. This resulted in 469 individuals assigned to the Caucasian group, 28 individuals assigned to the African descent or African American group, and 18 individuals assigned as “unknown”. The data set for further analysis was restricted to Caucasian samples.

*Age*. Ages were imputed using the Elastic Net method [47]. This method performs model selection and parameter estimation in a manner that is a combination of ridge-regression and the lasso. The prediction method is also explained in [47]. For computational reasons, λ was set to zero, in which case the Elastic Net method reduces to the lasso method. For most applications, experience demonstrates that the optimal value for λ is zero or quite near zero.

Ages were imputed using separate models for each data source, due to evidence of a source effect, and each sex separately. In cases where the sex was missing or the reported sex was different from the sex implied by the expression data, the sex implied by the expression data was used. This was done so that in the case the annotation data and expression data were mismatched, the imputed age would correspond to the data used to predict it.

The 5,000 genes with the highest correlation to age were used as potential regressors. Cross-validation was used to select the number of steps in the model selection procedure. The number of predictors in the model was between 67 and 76 for the four different models. The percentage of variation explained in the training set is quite high (97%–99%) for three of the models. For the fourth, the model for Vanderbilt females, the percentage of variation explained was slightly lower, 0.92. This is a vast improvement over more naïve imputation methods that are used when adjusting for covariates with missing data, where mean values of the nonmissing data are used to fill in the missing values. Very few of the predictors we constructed were common between the different models. Given the number of predictors with high correlation to age, this is not surprising. Nonetheless, within a given data source (i.e., Pittsburgh or Vanderbilt samples), the male model is a reasonable predictor for the ages of the females and vice-versa. This same trend did not hold for predicting the ages of same-sex individuals across data sources.

### Statistical and data visualization methods.

*Expression trait processing.* Expression traits were adjusted for age, sex, and medical center. Residuals were computed using rlm function from R statistical package (M-estimation with Tukey's bisquare weights). In examining the distributions of the mean log ratio measures for each expression trait in the HLC set, we noted a high rate of outliers. As a result, we used robust residuals and nonparametric tests to carry out the association analyses in the HLC. For each expression trait, residual values deviating from the median by more than three robust standard deviations were filtered out as outliers.

Genome-wide eQTL association analysis. The Kruskal-Wallis test was used to determine association between adjusted expression traits and genotypes. We chose this nonparametric method because of its robust nature to underlying genetic model and trait distribution. *p*-Values were computed using nag_mann_whitney (for loci with two observed genotypes) and nag_kruskal_wallis_test (for loci with three observed genotypes) routines from NAG C library (http://www.nag.co.uk). We used FDR for multiple-test correction. FDR was estimated as the ratio of the average number of eQTLs found in datasets with randomized sample labels to the number of eQTLs identified in the original data set. Since the number of tests was large (∼1,010), we found the empirical null distribution was very stable and three permutation runs were sufficient for convergence to estimate FDR. FDR computation was performed separately for *cis* (<1 Mb probe to SNP distance) and *trans* associations resulting in nominal *p*-value cutoffs of 5.0 × 10^−5^ and 1.0 × 10^−8^ for *cis* and *trans* eQTLs, respectively.

*Targeted set association analysis.* The 3,346 SNPs identified in the first round of analysis as associating with expression traits in *cis* at an FDR < 0.1 were picked for a second round of analysis. To assess the significance of the resulting set of expression traits detected as associated with this set of SNPs, sets of randomly selected SNPs of size 3,346 with MAF distributions identical to the original set were generated. All sets of SNPS were then analyzed using the same method described above for genome-wide associations.

*Identifying differentially expressed genes.* To assess whether a gene in a given sample was differentially expressed, we used a previously described and validated error model for testing whether the mean log ratio of the intensity measures between the experiment and reference channels was significantly different from zero [42,43,48]. Based on this error model we obtained *p*-values for each of the individual gene expression measures in each sample as previously described [33]. We then computed the standard deviation of –log_10_ of the *p*-value for each gene expression measure over all samples profiled for a given tissue, and then rank ordered all of the genes profiled in each tissue based on this standard deviation value (rank ordered in descending order). Genes that fall at the top of this rank ordered list can be considered as the most differentially expressed or variable genes in the study. We have previously shown that this type of ordering approach well captures the most active genes in a set of samples [33]. For demonstrating the number of genome-wide significant eQTLs and eSNPs as a function of differential gene expression, we binned the expression traits into quartiles (Q1-Q4) based on the rank-ordered gene list, with each bin containing 10,025 genes and the bins increasing in significance with respect to differential expression, from Q1 to Q4.

*Visualization of networks.* Networks were visualized using the Target Gene Information (TGI) Network Analysis and Visualization (NAV) desktop application developed at Rosetta Inpharmatics. This tool enables rapid, real-time, graphical analysis of pathway network models built from a comprehensive and fully integrated set of public and proprietary interaction databases available through a back-end central database, described in detail in a separate report. Additionally, the TGI NAV tool supports experimentally generated systems biology data such as the statistical associations and causal relationships described here. TGI NAV enables integration and visualization of orthogonal data sets using network models as a framework and facilitates dissection of networks into smaller, functionally significant subnetworks amenable to biological interpretation.

To construct the local networks for *H2-Eb1*, *Erbb3*, and *Rps26*, the whole-gene probabilistic causal networks were loaded into the database and the TGI NAV tool was used to extract all edges from this network involving the central gene of interest. In the case of the *Erbb3* network, the local network was expanded by extracting all additional edges involving any genes directly connected to *Erbb3*. Note that while the underlying networks describe causal relationships between transcripts, TGI NAV was used to translate this network into the space of genes using an integrated mapping database that clusters transcripts into gene models utilizing their genomic coordinates. As a result, multiple causal relationships between gene pairs can be observed in cases where multiple transcripts for a single gene were profiled. Visualization properties of nodes (e.g., color) are specified in TGI NAV either for individual nodes, or in a data-driven manner by associating attributes, such as KEGG pathway membership, with groups of nodes and mapping visualization properties to these attributes.

## Supporting Information

Figure S1 Atlas of Gene Expression for *Rps26* and *Erbb3*
For all panels, the horizontal bar for each row represents the mean expression value and the horizontal line indicating the standard deviation. The red arrow off to the left highlights the pancreas tissue.
(A) Expression levels of *Rps26* in 60 murine tissues and cell lines. The tissues and cell lines are given along the *y*-axis, and the mean relative transcript abundances are given along the *x*-axis.
(B) Expression levels of *Erbb3* in 60 murine tissues and cell lines. The tissues and cell lines are given along the *y*-axis, and the mean relative transcript abundances are given along the *x*-axis.
(C) Expression levels of *Rps26* in 46 monkey tissues and cell lines. The tissues and cell lines are given along the *y*-axis, and the mean relative transcript abundances are given along the *x*-axis.
(D) Expression levels of *Erbb3* in 46 monkey tissues and cell lines. The tissues and cell lines are given along the *y*-axis, and the mean relative transcript abundances are given along the *x*-axis.
(E) Expression levels of *RPS26* in 50 human tissues and cell lines. The tissues and cell lines are given along the *y*-axis, and the mean relative transcript abundances as determined by each of six individual reporters on the microarray that target RPS26 are given along the *x*-axis.
(F) Expression levels of *Erbb3* in 50 human tissues and cell lines. The tissues and cell lines are given along the *y*-axis, and the mean relative transcript abundances are given along the *x*-axis.
(441 KB PDF)

Table S1 Population Demographics of the HLC (81 KB XLS)

Table S2 Association Results for HLC Expression and Genotyping Data (1.64 MB XLS)

Table S3 Expression Traits Corresponding to Genes Associated with Human Diseases Are under Significant Genetic Control in the HLC (172 KB DOC)

Table S4 Genes Represented on the HLC Microarray Described in the Main Text (7.97 MB XLS)

Table S5 Genes Represented on the BXH/apoE Microarray Described in the Main Text (9.33 MB XLS)

Table S6 Genes Represented on the BXH/wt and BXC Microarray Described in the Main Text (6.58 MB XLS)

### Accession Numbers

All microarray data associated with the HLC have been deposited into the Gene Expression Ominbus database under accession number GSE9588.

## Abbreviations

CAD: coronary artery disease
eQTL: expression quantitative trait locus
ER: endoplasmic reticulum
FDR: false discovery rate
GWAS: genome-wide association study
HLC: human liver cohort
LD: linkage equilibrium
MEM: macrophage-enriched metabolic
QTL: quantitative trait locus
SNP: single nucleotide polymorphism
T1D: type 1 diabetes
WTCCC: Wellcome Trust Case Control Consortium

## Note Added in Proof

Reference 49 is cited out of order in the article because it was added during the proof stage.
