# Supplementary material for: Mapping the Genetic Architecture of Gene Expression in Human Liver
Source: PLoS Biol. 2008 May 6;6(5):e107. doi: 10.1371/journal.pbio.0060107 (PMC2365981; doi:10.1371/journal.pbio.0060107)

A)

Rps26 Expression in Mouse

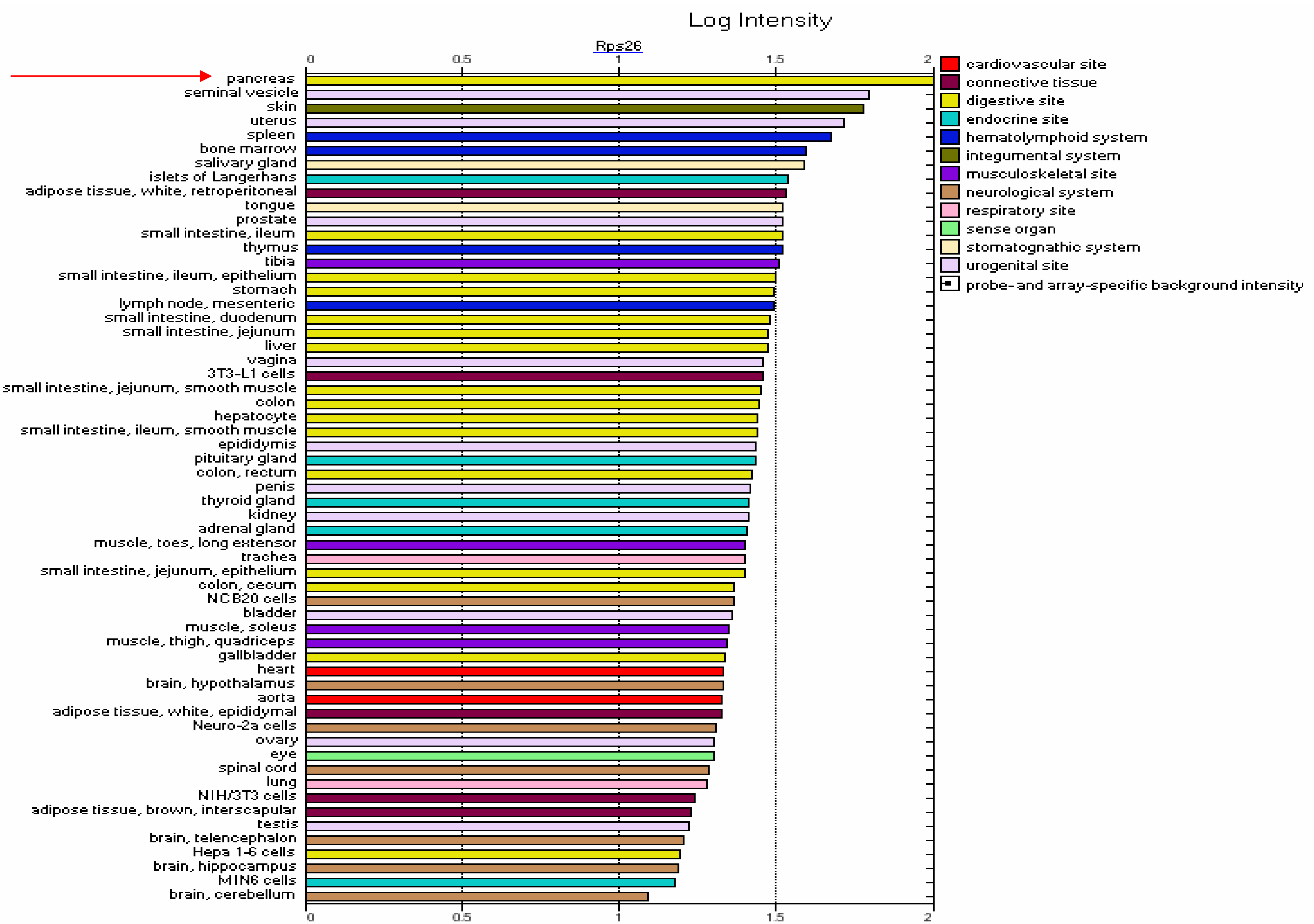

B)

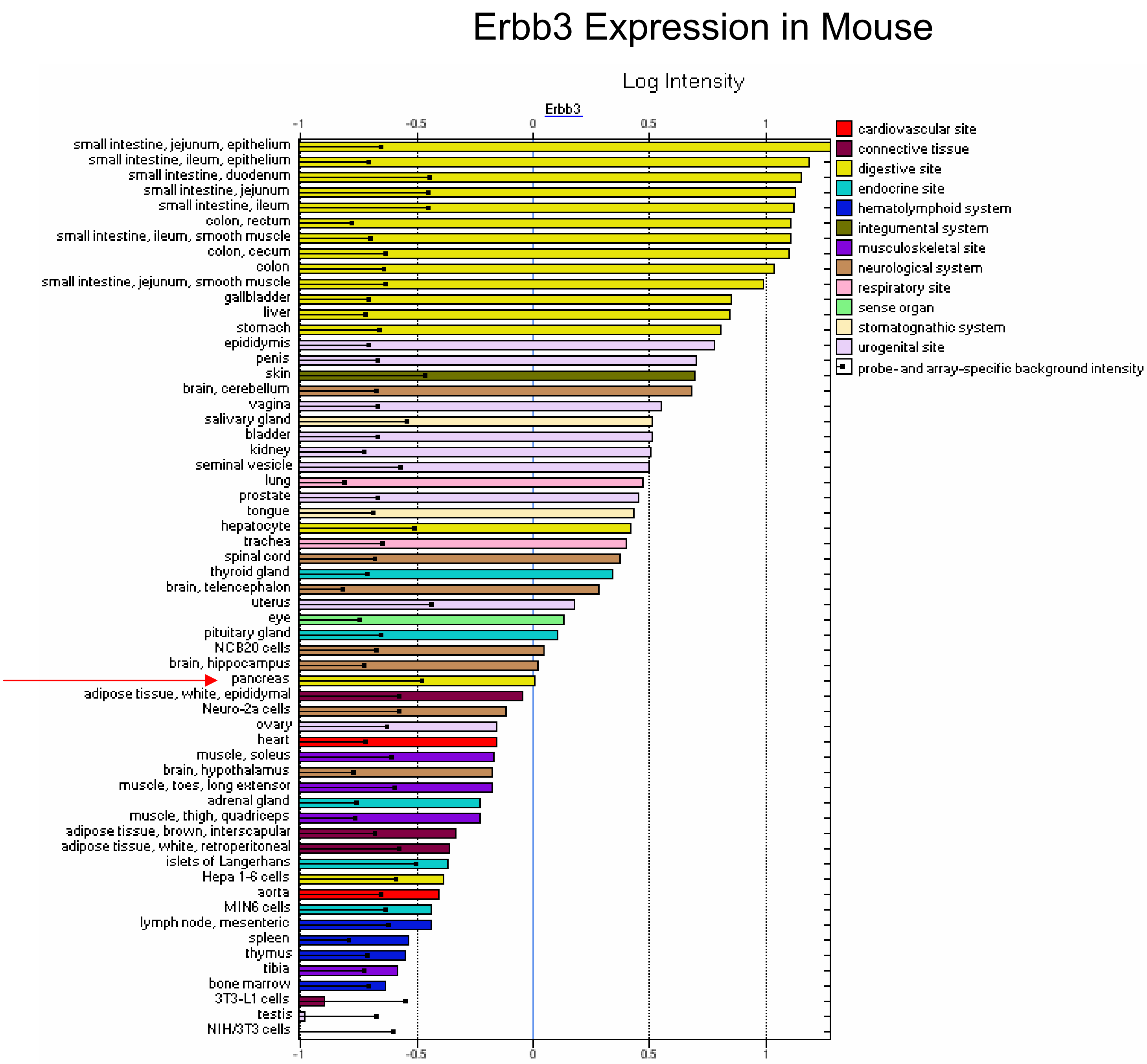

Erbb3 Expression in Mouse
Log Intensity
Erbb3
-1
-0.5
0
0.5
1
small intestine, jejunum, epithelium
small intestine, ileum, epithelium
small intestine, duodenum
small intestine, jejunum
small intestine, ileum
colon, rectum
small intestine, ileum, smooth muscle
colon, cecum
colon
small intestine, jejunum, smooth muscle
gallbladder
liver
stomach
epididymis
penis
skin
brain, cerebellum
vagina
salivary gland
bladder
kidney
seminal vesicle
lung
prostate
tongue
hepatocyte
trachea
spinal cord
thyroid gland
brain, telencephalon
uterus
eye
pituitary gland
NCB20 cells
brain, hippocampus
pancreas
adipose tissue, white, epididymal
Neuro-2a cells
ovary
heart
muscle, soleus
brain, hypothalamus
muscle, toes, long extensor
adrenal gland
muscle, thigh, quadriceps
adipose tissue, brown, interscapular
adipose tissue, white, retroperitoneal
islets of Langerhans
Hepa 1-6 cells
aorta
MIN6 cells
lymph node, mesenteric
spleen
thymus
tibia
bone marrow
3T3-L1 cells
testis
NIH/3T3 cells
cardiovascular site
connective tissue
digestive site
endocrine site
hematolymphoid system
integumental system
musculoskeletal site
neurological system
respiratory site
sense organ
stomatognathic system
urogenital site
probe- and array-specific background intensity

C)

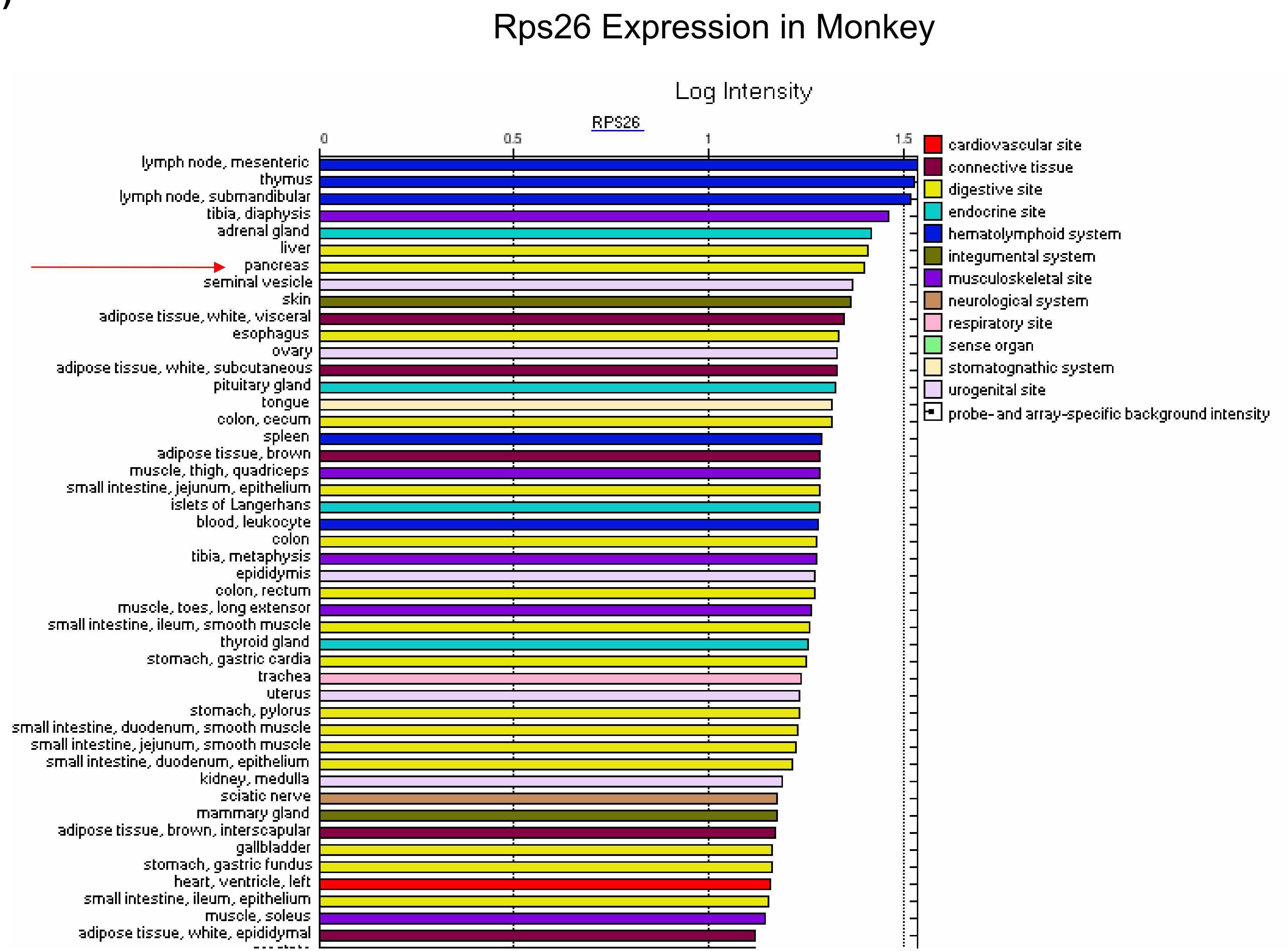
Rps26 Expression in Monkey
Log Intensity
RPS26
0
0.5
1
1.5
lymph node, mesenteric
thymus
lymph node, submandibular
tibia, diaphysis
adrenal gland
liver
pancreas
seminal vesicle
skin
adipose tissue, white, visceral
esophagus
ovary
adipose tissue, white, subcutaneous
pituitary gland
tongue
colon, cecum
spleen
adipose tissue, brown
muscle, thigh, quadriceps
small intestine, jejunum, epithelium
islets of Langerhans
blood, leukocyte
colon
tibia, metaphysis
epididymis
colon, rectum
muscle, toes, long extensor
small intestine, ileum, smooth muscle
thyroid gland
stomach, gastric cardia
trachea
uterus
stomach, pylorus
small intestine, duodenum, smooth muscle
small intestine, jejunum, smooth muscle
small intestine, duodenum, epithelium
kidney, medulla
sciatic nerve
mammary gland
adipose tissue, brown, interscapular
gallbladder
stomach, gastric fundus
heart, ventricle, left
small intestine, ileum, epithelium
muscle, soleus
adipose tissue, white, epididymal
cardiovascular site
connective tissue
digestive site
endocrine site
hematolymphoid system
integumental system
musculoskeletal site
neurological system
respiratory site
sense organ
stomatognathic system
urogenital site
probe- and array-specific background intensity

D)

Erbb3 Expression in Monkey

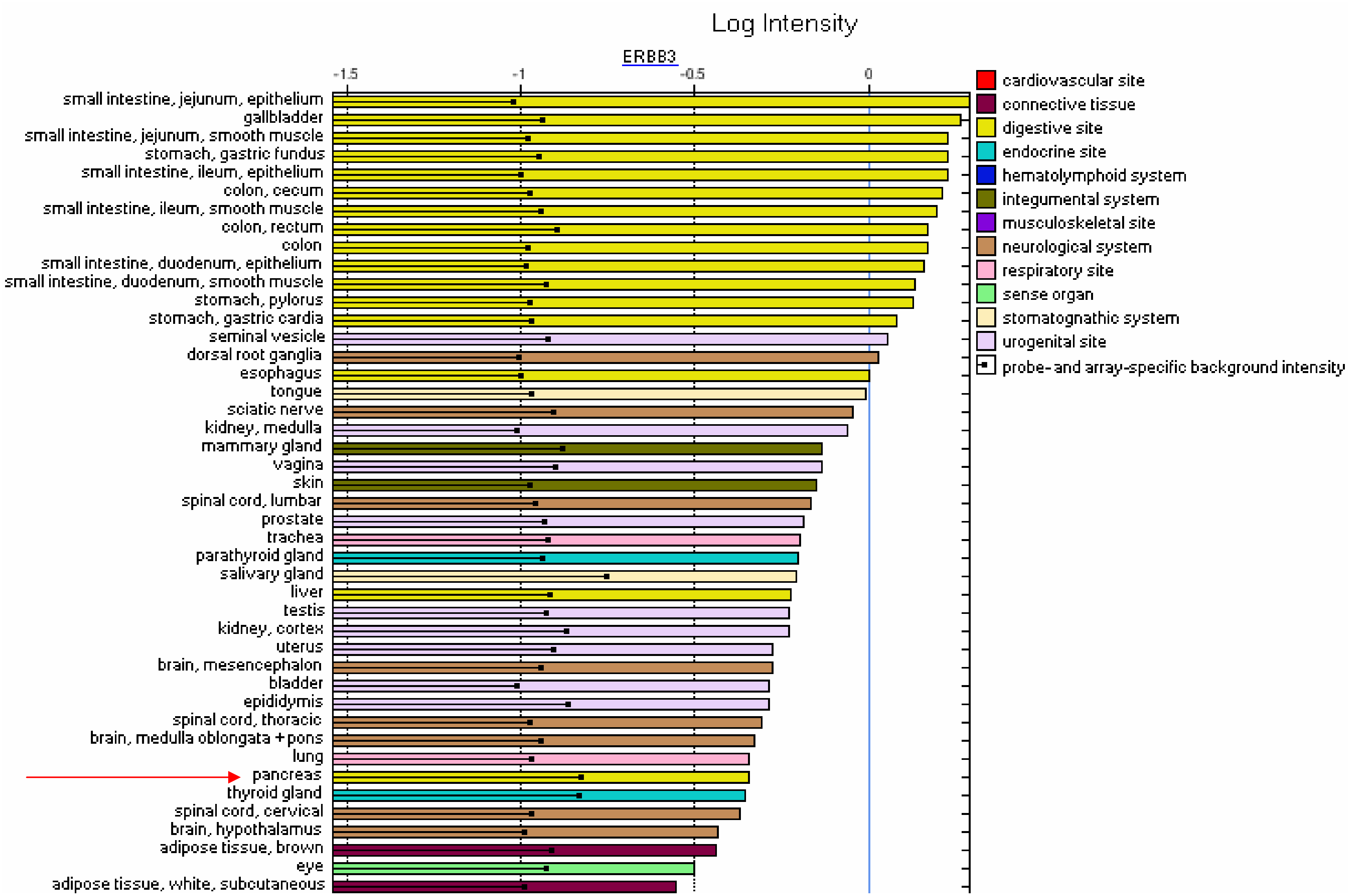

E)

Rps26 Expression in Human

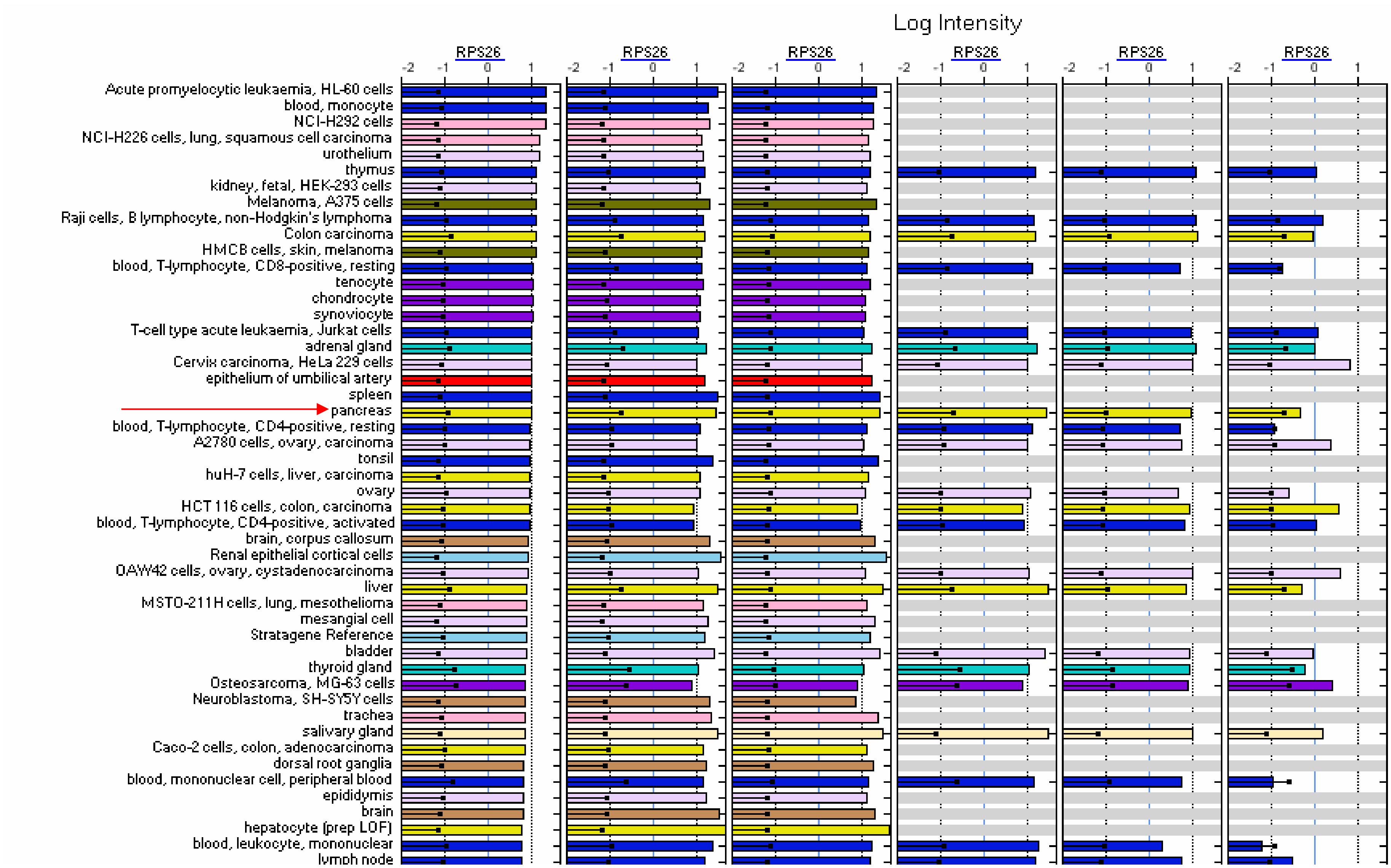

F)

Erbb3 Expression in Human

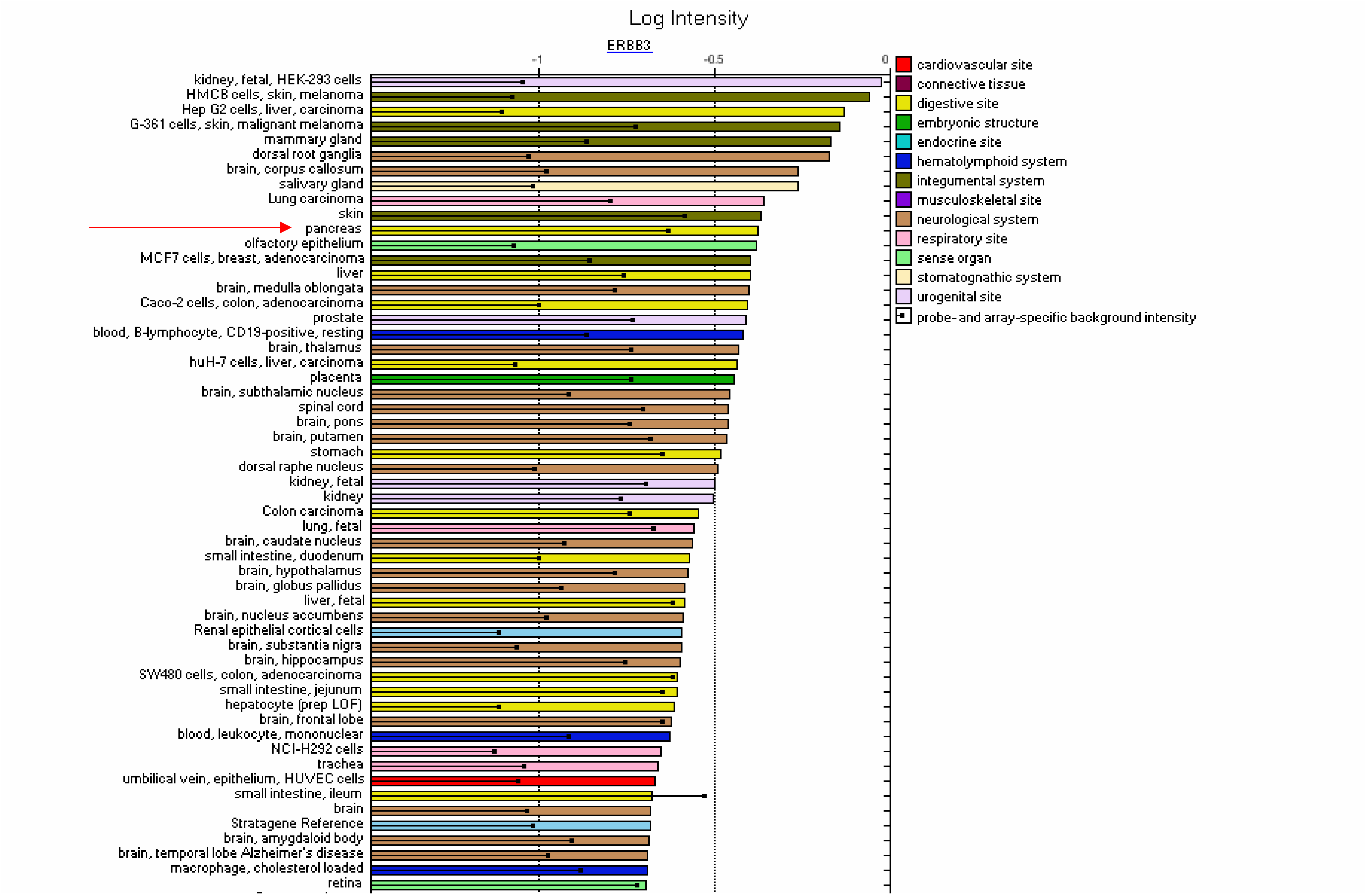

Supplement: Supplementary file 1 [file pbio.0060107.sg001.pdf]
