# Supplementary material for: Mapping the Genetic Architecture of Gene Expression in Human Liver
Source: PLoS Biol. 2008 May 6;6(5):e107. doi: 10.1371/journal.pbio.0060107 (PMC2365981; doi:10.1371/journal.pbio.0060107)
Supplement: Supplementary file 2 [file pbio.0060107.st003.doc]

**Supplementary Table 3.** Expression traits corresponding to genes associated with human diseases are under significant genetic control in the human liver cohort.

| **Gene Symbol** | **Associated Disease** | **Gene Location**† | **eSNP ID** | **eSNP Location**† | **eSNP Association**  **P-value**‡ |
| --- | --- | --- | --- | --- | --- |
| ABCG5 | Cholesterol/Plant Sterols[11] | 2:43,893,115 | rs3792009 | 2:43,948,984 | $1.58\times10^{-7}$ |
| ATP7B | Wilson Disease-Associated Protein[12] | 19:50,141,082 | rs4943046 | 13:51,417,747 | $3.24\times10^{-10}$ (*) |
| BRCA1 | Breast Cancer[13] | 17:38,449,839 | rs8070085 | 17:38,595,510 | $9.73\times10^{-17}$ |
| CARD15 | Inflammatory Bowel Disease[14,15] | 16:49,288,550 | rs1981760 | 16:49,280,575 | $1.90\times10^{-16}$ |
| CFH | Age-Related Macular Degeneration[16-18] | 1:194,887,763 | rs7542235 | 1:193,555,270 | $6.94\times10^{-22}$ |
| CLOCK | Circadian Rhythm[19] | 4:55,993,416 | rs1777642 | 4:56,028,303 | $4.05\times10^{-5}$ |
| EVC | Ellis van Creveld Syndrome[20] | 4:5,763,824 | rs4513510 | 4:5,839,846 | $5.12\times10^{-15}$ |
| HPS1 | Hermansky-Pudlak Syndrome[21] | 10:100,165,945 | rs11189600 | 10:100,169,264 | $2.44\times10^{-5}$ |
| LRRK2 | Parkinson’s Disease[22,23] | 12:39,047,977 | rs10878123 | 12:38,818,800 | $4.82\times10^{-6}$ |
| NRG1 | Schizophrenia[24] | 8:32,624,285 | rs10503915 | 8:32,404,719 | $1.83\times10^{-5}$ |
| PAH | Phenylketonuria[25] | 12:101,756,234 | rs11111760 | 12:10,666,365 | $6.55\times10^{-7}$ |
| PCSK9 | Heart Disease[26] | 1:55,277,807 | rs10888843 | 1:54,401,482 | $7.33\times10^{-4}$ |
| PPARA | Type 2 Diabetes[27] | 22:44,996,584 | rs243303 | 14:32,805,179 | $6.65\times10^{-10}$(*) |
| PSEN2 | Alzheimer’s Disease[28-31] | 1:225,125,582 | rs1800672 | 1:223,392,197 | $6.23\times10^{-10}$ |
| SQSTM1 | Paget Disease of Bone[32] | 5:179,180,502 | rs565280 | 5:179,186,532 | $2.30\times10^{-20}$ |
| TGFBR2 | Obesity[33] | 3:30,622,997 | rs7625163 | 3:31,150,505 | $1.25\times10^{-5}$ |
| USF1 | Familial Combined Hyperlipidemia[34] | 1:159,275,665 | rs12118043 | 1:158,378,491 | $2.73\times10^{-5}$ |
| VDR | Asthma[35,36], Cancer[37] | 12:46,521,588 | rs2239182 | 12:46,541,678 | $3.59\times10^{-5}$ |
| VKORC1 | Warfarin Dosing[38] | 16:31,009,676 | rs4889606 | 16:30,918,684 | $1.66\times10^{-23}$ |
| WRB | Congenital Heart Disease[39] | 21:39,674,139 | rs2837006 | 21:39,694,306 | $3.68\times10^{-21}$ |
| ZFP90 | Obesity[33] | 16:67,157,380 | rs6505772 | 18:12,924,029 | $4.54\times10^{-9}$(*) |

†Gene and SNP coordinates are given with respect to NCBI Build 36 of the human genome. Gene position reflects the start coordinate of the gene as defined in the UCSC Genome Browser Database.

‡Nominal p-values represent the significance of the association between SNP genotypes and gene expression measures for the indicated SNP and gene, respectively, as defined in the text. These p-values are associated with FDRs < 0.10.

*Indicates that the association was considered trans-acting, as defined in the text. All other associations reported are cis-acting.
